# Safely Targeting Cancer, the Wound That Never Heals, Utilizing CBP/Beta-Catenin Antagonists

**DOI:** 10.3390/cancers17091503

**Published:** 2025-04-29

**Authors:** Yusuke Higuchi, Jia-Ling Teo, Daniel Yi, Michael Kahn

**Affiliations:** 1 Beckman Research Institute, City of Hope, Duarte, CA 91010, USA; yhiguchi@coh.org; 2 Department of Cancer Biology and Molecular Medicine, Beckman Research Institute, City of Hope, Duarte, CA 91010, USA; jteo@coh.org (J.-L.T.); daniel.yi.hz@gmail.com (D.Y.)

**Keywords:** CBP, p300, β-catenin, somatic stem cell (SSC), cancer stem cell (CSC), fatty acid oxidation (FAO), glycolysis, fibrosis, cancer, quiescence

## Abstract

Stem cells, both normal somatic (SSC) and cancer stem cells (CSC) exist in minimally two states, i.e., quiescent and activated. Regulation of these two states, including their reliance on different metabolic processes, i.e., FAO and glycolysis in quiescent versus activated stem cells respectively, involves the analysis of a complex array of factors (nutrient and oxygen levels, adhesion molecules, cytokines, etc.) to initiate the epigenetic changes to either depart or enter quiescence. Quiescence is a critical feature of SSC that is required to maintain the genomic integrity of the stem cell pool, particularly in long lived complex organisms. Quiescence in CSC, whether they are derived from mutations arising in SSC, aberrant microenvironmental regulation, or via dedifferentiation of more committed progenitors, is a critical component of therapy resistance and disease latency and relapse. At the beginning of vertebrate evolution, approximately 450 million years ago, a gene duplication generated the two members of the Kat3 family, CREBBP (CBP) and EP300 (p300). Despite their very high degree of homology, these two Kat3 coactivators play critical and non-redundant roles at enhancers and super-enhancers via acetylation of H3K27, thereby controlling stem cell quiescence versus activation and the cells metabolic requirements. In this review/perspective, we discuss the unique regulatory roles of CBP and p300 and how specifically targeting the CBP/β-catenin interaction utilizing small molecule antagonists, can correct lineage infidelity and safely eliminate quiescent CSC.

## Simple Summary

Unresolved wound healing is a critical factor in cancer. More generally, inappropriate wound healing can promote chronic progressive fibroinflammatory diseases, including organ fibrosis and neurodegeneration. Transient loss of lineage fidelity is important for wound healing however, persistent lineage infidelity is associated with cancer and fibrosis. CBP/β-catenin antagonists can safely target stem cells to induce differentiation and restore lineage fidelity to treat cancer, fibrosis and neurodegenerative diseases.

## 1. Introduction

Rudolf Virchow in 1858 first proposed the concept that “cancer is a wound that never heals” [1]. More recently the similarities between cancer and wounds have reemerged as a topic of investigation [2,3]. Patients with chronic wounds have increased cancer susceptibility [4,5] and in mice, mutations that retard the activation of stem cells, display reduced efficiency in wound closure, with increased cancer resistance [6].

Tissue resident adult mammalian somatic stem cells (SSC) are responsible for both homeostasis and wound repair. The accumulation of mutations in normal SSC that causes them to lose their homeostatic balance, favoring self-renewal with subsequent tissue overgrowth at the expense of differentiation, is associated with tumorigenesis. A transient growth versus differentiation imbalance is created during wound repair. In SSC, Wnt signaling contributes to wound repair and tissue regeneration that is hijacked by cancer stem cells (CSC) [7,8,9]. Stem cell lineage infidelity occurs transiently in wounds, however it persists in cancer [3]. Cellular plasticity and lineage infidelity are associated with differential super-enhancer (SE) activation and critical in both wound repair and malignancies [10,11,12]. Multiple factors contribute to stem cell lineage infidelity, including cellular metabolism, hypoxia, inflammation and mechanical features of the tumor microenvironment (TME) [13]. Stem cell lineage infidelity and the epigenetic reprogramming associated with it, mediate immune escape in CSC [13,14]. Tumor-intrinsic Wnt/β-catenin signaling is associated with cancer immune evasion, immunosuppressive cell subsets, defective dendritic cell presentation and T-effector cell recruitment [15,16]. Wnt signaling is critical in stem cell biology and the lineage infidelity associated with it in wound healing and cancer [17,18,19], fibrosis [20] and neurodegeneration [21].

Aging effects Wnt/β-catenin signaling [22,23] and significantly negatively impacts healing by prolonging the inflammatory phase, which can lead to chronic wound healing [24]. Age-related lineage infidelity or precocious differentiation has been seen in various tissues, related to both intrinsic changes in SSC and to the stem cell niche microenvironment [25,26,27,28]. The role of cellular plasticity and lineage infidelity in an array of diseases, which is further exacerbated with aging, highlights the importance of developing therapies to safely manipulate endogenous “stemness” (both normal and cancerous) and correct lineage infidelity via modulation of Wnt/β-catenin-regulated transcription [22,29,30].

## 2. Discussion

### 2.1. Wnt Signaling in Wound Healing

There are three important components to wound healing: epithelial movement, cell proliferation, and contraction (or remodeling) [31]. Wounding activated Wnt signaling is important in all stages of the healing process, including stem cell mobilization within the wound site, the control of inflammation, and remodeling [32]. At the wound edge, cells dedifferentiate to repair the wound [33,34,35]. The Wnt/β-catenin, Hippo/YAP-TEAD, TGF-β/Smad, SOX and Notch pathways can all play a role in the dedifferentiation of more committed cells as part of a regenerative program required for wound healing [10,36,37,38,39]. Blocking Wnt signaling impairs normal fin regeneration after amputation in zebrafish [40] and inhibiting Wnt signaling results in abrupt cessation of regeneration in animals with continually regenerating retinas [41,42,43,44]. Wnt signaling disruption blocks the recruitment of stem/progenitor cells to the wound [41,45,46,47] adversely affecting the proliferative phase of the wound healing process [48,49]. Wnt signaling clearly has dichotomous roles in the inflammatory response to wounding, playing both pro- and anti-inflammatory roles [32]. Wnt signaling is critical in normal wound healing, however chronic injury can aberrantly prolong Wnt signaling, thereby increasing the risk of oncogenic transformation [50,51].

### 2.2. Stem Cells; Normal Somatic Stem Cells (SSC) and Cancer Stem Cells (CSC)

With aging, significant deterioration in stem cell functionality occurs, leading to reduced (e.g., thinning of the epidermis and dermis) and/or aberrant tissue regeneration (e.g., fibrosis), increased degenerative diseases of the brain [52] (e.g., Alzheimer’s disease), skeletal muscle [53], skin [54,55] and cancer [56]. A limited pool exists of essentially immortal, generally quiescent SSC that serve as a reservoir for tissue regeneration. SSC exhibit inherent plasticity and exist in different states, minimally, quiescent versus activated. Infrequently entering the cell cycle during homeostasis, upon injury to repair damaged tissue, long-lived quiescent SSC do so more frequently. Through acquired mutations, SSC can become CSC, representing a “dark side” to their immortality. Similar to SSC, CSC can self-renew and differentiate, thereby maintaining, renewing and propagating tumors. Therapy resistance, disease relapse, and metastasis constitute the central challenges to more effective cancer therapy. The ability to eliminate CSC is the key to overcoming these challenges [57]. CSC may derive from long lived quiescent SSC. However, they can also originate from more differentiated cells via dedifferentiation to become CSC, particularly within the context of wounding and inflammation [58,59]. For example, the combination of oncogenic Kras and the activation of NF-κB, strongly activates Wnt signaling in intestinal epithelial cells (IEC), inducing dedifferentiation to a progenitor/stem cell-like state to generate stem-like IEC [60]. Niche inflammatory signals protect stem cells from cytotoxic stress and prevent differentiation via NF-κB induced Wnt signaling [61]. Cells with stem cell-like properties can also be generated via epithelial to mesenchymal transition (EMT) [62]. The tumor microenvironment via secretion of TGF-β inducing ZEB1 expression drives an EMT transition, critical for the conversion of non-CSC to CSC and the maintenance of the CSC-like state [63]. Secretion of hepatocyte growth factor by myofibroblasts inducing nuclear translocation of β-catenin activates Wnt signaling, thereby generating stem-like features in more differentiated colorectal tumor cells. This demonstrates that stromal cells by secreting extrinsic factors can activate Wnt signaling, creating a microenvironment that supports the dedifferentiation of colon cancer cells to a CSC phenotype [64].

CSC have far more similarities than differences to normal SSC, greatly complicating the safe elimination of CSC, regardless of how they arose. SSC and CSC express similar “stemness” markers and both reside in specialized niches making elimination of this cell population extremely difficult without deleterious effects to normal SSC [65,66,67].

### 2.3. Stem Cell Modes of Division and Implications

**Figure 1:**
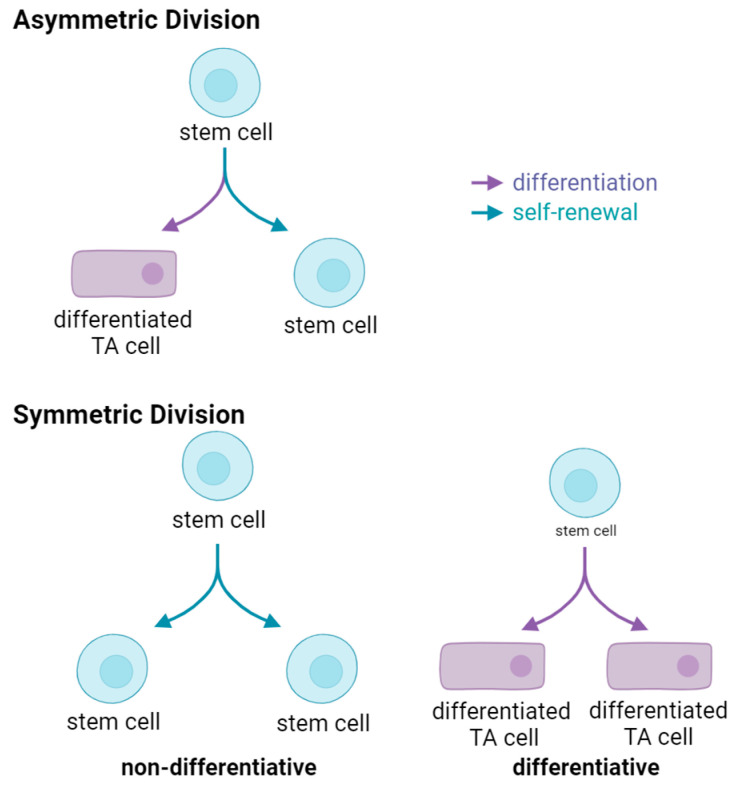
Modes of Stem Cell Division. Upper, an asymmetric division results in the production of two daughter cells with different fates—one remains a stem cell, the other becomes a differentiated transient amplifying (TA) cell. Lower left, symmetric non-differentiative division generates to two daughter stem cells. Lower right, symmetric differentiative division produces two differentiated transient amplifying daughter cells.

Rarely dividing [68,69] during normal tissue homeostasis, long-term SSC spend the majority of their lifetime in a quiescent state. Once activated, quiescent SSC enter the cell cycle and undergo mitosis, generating two daughter cells. Two basic modes of stem cell division exist, i.e., symmetric or asymmetric (Figure 1). Asymmetric division, whereby one daughter cell remains in its niche, while the other daughter proceeds forward initiating a differentiation process to maintain tissue homeostasis is ideal (Figure 1, upper panel). However, SSC can also undergo two modes of symmetric divisions. Symmetric non-differentiative divisions generate two daughter stem cells that remain in their niche, whereas in symmetric differentiative divisions both cells leave their niche and differentiate, and lose their “stemness”, (Figure 1, lower panel). Both modes of symmetric division are considered deleterious to the pool of long-lived SSC. Symmetric differentiative division leads to premature exhaustion of the stem cell pool [70], whereas symmetric non-differentiative division increases the number of DNA mutations accumulated in the SSC pool, a feature associated for example with Clonal Hematopoiesis of Indeterminate Potential (CHIP) [71].

Normal tissue homeostasis requires timely activation and asymmetric division of the SSC, however, with aging, this process is corrupted due to the accumulation of mutations in the SSC pool, chronic or acute injury, reversion of differentiated daughters to SSC, serum factors, changes in the niche microenvironment and SSC senescence [72]. The activation of quiescent SSC, and subsequent asymmetric versus symmetric division, is the most critical cellular decision in adult organisms underlying diseases of aging, including impaired wound healing, sarcopenia, fibrosis and osteoporosis, cancer and neurodegeneration. Stem cells (either normal SSC or CSC) undergoing mitosis read and must integrate an enormously complex array of information from their niche microenvironment to arrive at what in principle is a simple binary decision [29]. A fundamental intrinsic difference between CSC (and even pre-CSC for example in CHIP or myelodysplastic syndrome (MDS)) and normal SSC, is that CSC preferentially divide symmetrically rather than asymmetrically. Mutations in the gene p53 in breast cancer stem cells induce preferential symmetric cell division [73]. Premature exhaustion of normal hematopoietic stem cells (HSC) (due to increased symmetric differentiative divisions), with expansion of the leukemic stem cell (LSC) population due to increased symmetric non-differentiative divisions, is associated with loss of function of the tumor suppressor PTEN [74]. Genetic activation of Hedgehog signaling via indirect perturbation of Notch signaling, causes increased neural stem cell (NSC) symmetric divisions [75]. Preferential symmetric non- differentiative versus symmetric differentiative division is another intrinsic difference between CSC and normal SSC due to critical mutations (i.e., p53, p73, PTEN, etc.) or aberrant Wnt mediated mTOR activation [74,76]. However, in some instances, symmetric differentiative division may provide a mechanism to eliminate defective SSC [77].

### 2.4. Stem Cell Heterogeneity

Somatic stem cells are heterogeneous and exist minimally in two distinct states. When activated deeply quiescent SSC in G_0_ transition into a so-called G_alert_ phase before becoming fully activated and entering the cell cycle [78,79]. Although it is widely accepted that quiescent stem cells are arrested in G_0_, quiescent neural stem cells (NSC), at least in vitro, are found arrested in either G_0_ or G_2_, where G_2_ quiescent NSC reactivate prior to the activation of G_0_ NSC [80]. After injury, organismal survival often requires rapid tissue repair. Under these conditions strict stem cell hierarchy is often compromised with the occurrence of cellular plasticity, fate conversion, lineage infidelity and “dedifferentiation” and reacquisition of “stem cell” characteristics [30,81]. For example, minimally two distinct intestinal stem cell (ISC) populations: Lgr5^+^ columnar basal cells (CBC) and a deeply quiescent, asymmetrically dividing, less radiosensitive, +4 Bmi1^+^ ISC, exist [82,83,84,85]. Due to their mode of chromosomal segregation [86], asymmetric cell division, deeply quiescent status and enhanced radio resistance, +4 Bmi1^+^ ISC appear to behave more like *bona fide* long-lived SSC than Lgr5^+^ CBC [84]. However, these populations are inter-convertible, with both possessing the capacity to generate all intestinal epithelial lineages [87,88]. Additionally, ISC that have a transcriptional profile similar to fetal intestinal stem cells that are important in intestinal regeneration after injury, termed “revival CSC”, have been described. They express high levels of *Anxa1*, *Clu* and *Sca1* and are characterized by high YAP signaling and increased TNF-α, TGF-β, INF-γ and NF-κB signaling [71,89,90,91]. Another recent report demonstrated that after chemical injury, reprogramming of Lgr5^−^ but Lgr4^+^ differentiated colonic epithelial cells, via Rspo3 induced Wnt signaling, is critical for epithelial regeneration [92]. In other stem cell populations including the lung epithelium and mammary gland [30,61] and in the hematopoietic system [93], similar situations exist. In hair follicles, both bulge cells (BC) and neighboring hair germ (HG) possess “stemness” features, BC and HG cells can both regenerate the seven distinct lineages of the hair follicle. Bulge cells normally generate HG cells under homeostatic conditions, however, after laser-ablation HG can replenish depleted bulge cells, [94]. Multiple pathways exist whereby cellular plasticity can induce a “stem-like” state from partially committed or differentiated cells, which has important implications for wound repair, chronic inflammation, tumorigenesis and aging [10,11,58,81]. Wound healing and inflammation play critical roles in cellular plasticity. Experiments in 1990 first demonstrated, that targeting oncogenic H-ras to murine differentiated epidermis generated papillomas preferentially at sites of irritation and wounding [95]. Introduction of a single oncogenic mutation, without inflammatory insult, only initiate intestinal tumor formation within one of the putative ISC populations (i.e., Lgr5/prominin/Bmi1 positive populations) [84,96,97], and targeting transient amplifying (TA) cells either had no effect or generated only microadenomas [96]. Cellular plasticity, associated with wounding and chronic inflammation, provides a mechanism whereby more differentiated progeny, with increased levels of DNA damage, can revert to a “stem-like” state. In the colon, the NF-kappa B (NF-κB) inflammatory pathway is dominant. Epidemiological studies have demonstrated that patients with chronic inflammation are predisposed to cancer. Non-steroidal anti-inflammatory drug administration decreases the incidence of colorectal cancer [98,99]. Immortalization of differentiated human cells by DNA viruses, including Epstein-Barr virus (EBV), Kaposi sarcoma-associated herpesvirus (KSHV), human papillomaviruses (HPV), hepatitis B virus (HBV), and more recently Merkel cell polyomavirus (MCPyV) and RNA viruses such as Hepatitis C Virus (HCV) and human T lymphotropic virus (HTLV-1) has been demonstrated [100]. Persistent chronic infection can lead to oncogenesis and induced dedifferentiation thereby generating cells with CSC characteristics. “Corruption” of the stem cell pool via chronic inflammation and viral infection significantly increases the risk of cancer.

Epigenetic landscape alterations in tissues occur after injury licensing normally restricted cell fate transitions. DNA methylation and histone modifications play critical roles in regulating this plasticity [11]. Rewiring of the epigenetic landscape and environmental perturbation after injury, allows for pathways that normally do not exist in embryonic or steady state adult tissues. Super-enhancers (SE) control the identity, lineage commitment and plasticity of adult SSC. SE represent a small fraction of total enhancers, which contain dense clusters (‘epicenters’) of transcription factor (TF) binding sites [12].Understanding the biology of therapy resistance is critical to attain complete cancer cures. CSC, regardless of their cell of origin, represent the major cause of therapy resistance, metastatic disease and relapse. As discussed above, the many shared features of CSC and SSC, their heterogeneity (i.e., quiescent versus activated), and plasticity (i.e., EMT, lineage infidelity), complicates the safe elimination of CSC [101].

A variety of nutrient sources, akin to dining at a Smorgasbord table, can be sampled by stem cells (germ line and somatic) and CSC to suit their “behaviors” and “life-styles” and match their metabolic requirements [102]. The tumor stromal microenvironment, including endothelial cells, fibroblasts and adipocytes, serves up an assortment of “dishes” including glucose, lactate, ketone bodies, fatty acids and glutamine, to heterogeneous CSC [103,104,105]. Quiescence, low metabolic activity, and a protective niche microenvironment better safeguard stem cells, both SSC and CSC, against the accumulation of DNA damage compared to more differentiated activated cells [106]. Regulation of metabolic balance and the synthesis of antioxidants maintains intracellular redox homeostasis in conjunction with CSC-specific metabolism. In long-lived stem cells, reactive oxygen species (ROS)-induced damage to nuclear and/or mitochondrial DNA leads to mutations and therefore must be avoided. Interestingly, low levels of ROS are maintained in quiescent stem cells, despite primarily utilizing mitochondrial fatty acid oxidation (FAO) and oxidative phosphorylation (OXPHOS) [102]. The primary energetic contributor to the maintenance of deeply quiescent SSC and CSC is FAO [107,108,109,110]. How do quiescent stem cells (normal or cancerous) avoid the generation of ROS, which is deleterious to genomic integrity and also maintenance of quiescence while utilizing FAO? Primordial oocytes, a long-lived, in humans more than 40 years, quiescent stem cells that primarily utilize FAO, maintain functionality and genomic integrity to produce healthy offspring. They maintain low mitochondrial activity with a minimum of ROS generation. They accomplish this by silencing of the expression of mitochondrial complex I (MC1). MC1 is responsible for the oxidation of NADH to NAD^+^ with the release and leakage of electrons, which when received by oxygen generate ROS. They bypass the need for MC1 by utilizing mitochondrial complex II (MC2). MC2 catalyzes the oxidation of FADH2 to FAD2^+^ thereby serving as an entry point for electrons [111]. This process, albeit less efficiently, supports ATP production without generating large amounts of ROS [112]. Interestingly, we recently reported that the most deeply quiescent subset of a therapy resistant population of CD34^−^CD38^−^ chronic myeloid leukemic stem cells (LSC) [113], utilize a similar approach to primordial oocytes to maintain quiescence [108].These LSC suppress the expression of MC1 complex genes, providing a mechanism to rely primarily on FAO while minimizing ROS. Pharmacologic differentiation of these quiescent LSC with ICG-001, a small molecule CBP/β-catenin antagonist, initiates the expression of MC1 genes and exit from quiescence. More generally, we believe that long lived quiescent stem cells rely on mitochondrial FAO, without complex I expression, thereby mitigating vulnerability to ROS. This allows deeply quiescent CSC, which are highly resistant to cancer chemo- and immunotherapy and a reservoir for disease recurrence and relapse, to persist for many years [114,115].

### 2.5. Wnt Signaling in Stem Cells; Low and High

The Wnt pathway is critical for the specification and maintenance of SSC in multiple tissues and organs, including the intestines, heart, blood, brain, and mammary gland [116]. Therefore, not surprisingly, a recurrent theme in cancer biology is the involvement of aberrant Wnt signaling [117,118]. In partnership with other key development pathways, Wnt regulation of SSC is important throughout development and organogenesis [119,120]. However, whether Wnt signaling is critical for self-renewal and maintenance of potency or stem cell differentiation and lineage commitment is debated [57]. The maintenance of ES cell pluripotency and the expansion of neural stem/progenitors is Wnt/β-catenin regulated [121,122]. However, ES cell differentiation [123] and lineage determination in neural crest stem cells is also controlled by Wnt/β-catenin signaling [124,125]. However, the dichotomous roles of Wnt/β-catenin signaling in stem cell biology is not adequately explained by the ‘Goldilocks’ concept of a “just right” level of nuclear Wnt/β-catenin transcription [126,127,128,129]. Similarly in cancer, a poorer prognosis is associated with increased Wnt/β-catenin signaling in colorectal tumors [130,131,132], however, increased nuclear β-catenin in tumors is associated with a more favorable melanoma prognosis [133,134,135,136]. However, a recent report outlined how a TCF4-dependent gene regulatory network conferred immunotherapy resistance in melanoma [137], consistent with previous reports of enhanced Wnt signaling in immunotherapy resistance [16,138].

### 2.6. Wnt Signaling and Metabolism

In stem cells, cellular and tissue differentiation and in immune cell biology, integration of cellular metabolism and cellular function is critical and highly evolutionarily conserved. In addition to playing a significant role in stem/progenitor cell biology [9,29,139], the Wnt signaling cascade regulates glucose metabolism, de novo lipogenesis and mitochondrial physiology [140,141], thereby providing a mechanism to couple metabolism with cellular status, i.e., quiescence, activation and differentiation [142]. The nuclear receptor (NR) family also plays essential regulatory roles in stem cell maintenance, differentiation and lineage commitment and metabolism [143]. Fatty acid oxidation (FAO), gluconeogenesis, lipogenesis and glycolysis are regulated by NR family members [144]. The nuclear receptors PPARα and ESRRA, in proximal tubule kidney cells, coordinate metabolism with differentiation. Their dysfunction is associated with kidney fibrosis [145]. Wnt signaling is also important in kidney cell differentiation [146], and aberrant Wnt activation and blocked differentiation with renal fibrosis [147]. Interferon activation of the signal transducer and activator of transcription 1 (STAT1)/Janus kinase (JAK) pathway, regulates immunometabolism and immune cell polarization [148]. Interferon also plays dichotomous roles in that it possesses antifibrotic effects via differentiation [149], yet also contributes to renal fibrosis and chronic kidney disease [150]. Wnt/β-catenin crosstalk and multiple signaling cascades converge at the amino-termini of the Kat3 coactivators to regulate metabolism and differentiation to maintain homeostasis or to resolve inflammatory processes after injury. Aberrant coordination is associated with cancer, fibrosis and neurodegeneration [22].

### 2.7. Differential Kat3 Coactivator Usage

**Figure 2:**
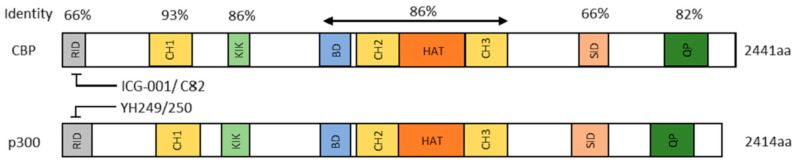
CBP and p300. An extremely high percentage of identity and even higher homology. between various regions of these large Kat3 coactivators at the amino acid level, despite their divergence over 450 million years ago. The CBP and p300 amino termini, the regions to which CBP/β-catenin (ICG-001 and C82 the active agent derived from PRI-724 dephosphorylation) and the direct p300/β-catenin antagonists (YH249, 250) bind respectively, are the most divergent regions.

β-catenin must recruit either cAMP response element-binding protein (CREB-binding protein (CREBPP or CBP) or its closely related homolog, p300 (E1A-binding protein, 300 KDa), in addition to the basal transcriptional apparatus to activate gene transcription [151,152,153]. Just prior to the vertebrate radiation, gene duplication gave rise to CBP and p300, very large proteins, both over 300 kD, encoded over 33 exons and 31 exons respectively [154]. Despite diverging over 450 million years ago, they retain an extremely high degree of identity, up to 93%, particularly over a large central core that includes the CH1, KIX, Bromodomain, and CH2 and CH3 regions (Figure 2) [155,156]. CBP and p300 are master orchestrators of transcription interacting with hundreds of proteins in this role. Perhaps not surprisingly, they are often considered redundant and interchangeable due to their high degree of protein sequence identity and even higher similarity. However, the retention of more than 150 Kb of redundant DNA for over 450 million years, is not something ‘Mother Nature’ would do without a very good reason! and numerous studies have clearly shown that CBP and p300 have definitive and unique roles both in vitro and in vivo [29,157,158,159,160]. Furthermore, the divergent roles of CBP and p300 are critical for the maintenance of fidelity in long-lived SSC (CBP) and the timely activation and proliferation of differentiated TA cells (p300) [29].

**Figure 3:**
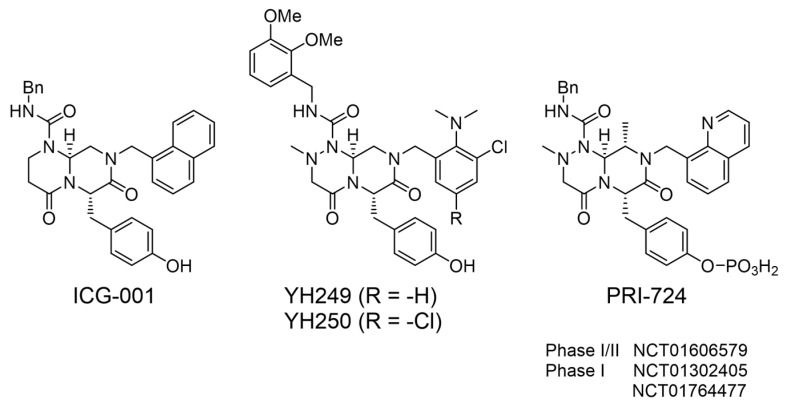
Structures of ICG-001, PRI-724 and YH-249/250.

Over 25 years ago, utilizing a cell-based TopFlash Wnt reporter assay in SW480 cells, with a library of 5000 secondary structure mimetics, my lab originally identified ICG-001 (IC_50_ ~3 μM) (Figure 3) [161]. We identified and validated that the molecular target of ICG-001 was the Kat3 coactivator CBP. With high affinity (~1 nM in vitro), ICG-001 binds the extreme N-terminus of CBP specifically, thereby directly inhibiting the CBP/β-catenin interaction. Critically important, ICG-001 does not bind to p300 [161,162]. We later identified the structurally related small molecule p300 direct binders, YH249 andYH250, which block p300/β-catenin driven transcription (Figure 3) [163].

The extreme N-termini of CBP and p300 are specifically and directly bound by ICG-001 and YH249/250 respectively. These regions of CBP and p300, with only 66% identity between them, are the least conserved regions (Figure 2). It appears that after initial evolutionary divergence of these N-terminal regions, within the individual N-terminal regions of both CBP and p300, >98% identity has been retained at the amino acid level for more than 100 million years! This confirms the crucial roles that these evolutionarily conserved regions have in vertebrate biology.

**Figure 4:**
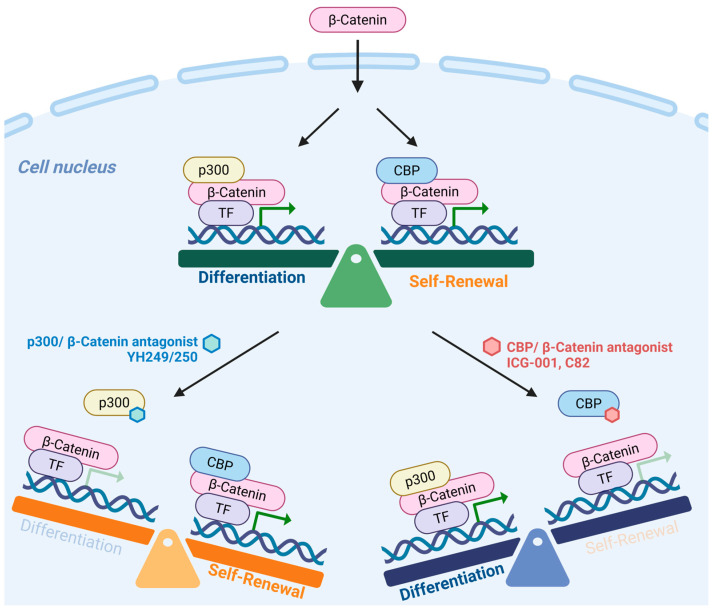
CBP/β-catenin antagonists ICG-001/C82 selectively block the CBP/β-catenin interaction leading to enhanced p300 usage thereby initiating differentiation. YH-249/250 selectively block the p300/β-catenin interaction, thereby enhancing CBP usage, resulting in maintenance of a program supporting self-renewal.

Selectively blocking the CBP/β-catenin interaction leads to enhanced p300/β-catenin transcription, resulting in the initiation of differentiation in a wide variety of stem/progenitor cells, including CSC [113,164,165,166,167]. Directly or indirectly blocking the p300/β-catenin interaction enhances CBP/β-catenin transcription in both mouse and human ES, iPS, and SSC, both in vitro and in vivo, which is critical for stem cell self-renewal [163,164,168,169,170]. These studies led to the development and validation of our model, in which CBP/β-catenin maintains stemness/enhances and self-renewal and p300/β-catenin transcription initiates a differentiative program, providing distinct roles for CBP/ and p300/β-catenin-mediated transcription in stem/progenitor cell biology (Figure 4) [29,167,168].

### 2.8. CBP/β-Catenin Antagonists Are Safe and Efficacious

CBP/β-catenin antagonists have been extensively investigated in a variety of preclinical tumor models. CBP/β-catenin antagonists safely eliminate quiescent drug-resistant CSC, via forced stochastic symmetric differentiation, without deleterious effects to normal endogenous SSC [77,113,171,172,173,174]. CBP/β-catenin antagonists have proven efficacious in a wide array of disease and injury models; including pulmonary, renal, hepatic and systemic fibrosis [175,176,177,178], myocardial infarction [179], neuro-development and neuro-degeneration [77,167]. The beneficial effects of CBP/β-catenin antagonists in these preclinical models, are associated with enhanced activation and subsequent asymmetric differentiation of SSC, thereby correcting lineage infidelity and initiating proper repair and healing [29,129,180].

Based on these studies, PRI-724 (IC50 ~150 nM) (Figure 3), a second-generation CBP/β-catenin specific antagonist was developed and proved extremely safe both pre-clinically and clinically. The non-adverse event level in IND enabling toxicology studies in dogs, given 28-day continuous infusion of PRI-724, was 120 mg/kg/day. Plasma concentrations roughly 300 times the IC_50_ were maintained for 28 days. In a first-in-human clinical trial in cancer patients, PRI-724 was administered by 7 days of continuous i.v. infusion, with dose escalation from 40 to 1280 mg/m^2^/day. No dose limiting toxicities were observed. Reduction of the biomarker *survivin/BIRC5* with upregulation of the differentiation antigen *CK20* in circulating tumor cells (CTC), strongly correlated with plasma concentrations of drug (R = 0.97) [181], demonstrating on target efficacy. Likely due to the critical role of the highly conserved CBP N-terminus in stem cell maintenance, resistance to the PRI-724 was not observed [172]. PRI-724, in HCV-induced cirrhosis patients, for which no currently approved treatment exists, demonstrated encouraging anti-fibrotic activity, [182]. However, the lack of oral bioavailability of PRI-724 hampered its development. Eisai developed a next generation orally available analog E7386, that is purportedly a CBP/beta-catenin antagonist, however, comparative analysis of E7386 with the highly specific bona fide CBP/β-catenin antagonists, ICG-001 and C82 (the active agent derived from PRI-724) cast significant doubt that the mechanism of action of E7386 is via specific CBP/β-catenin antagonism [183].

**Figure 5:**
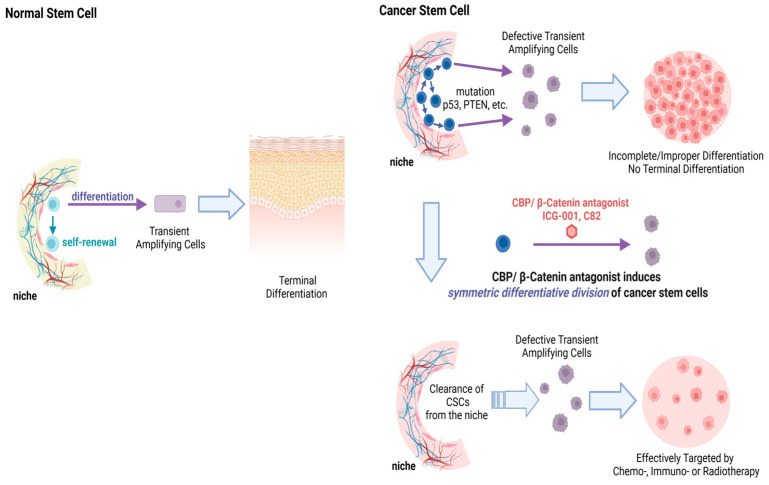
**Upper panel** Normal somatic stem cells (SSC) undergo asymmetric divisions—with the original stem cell remaining in the niche and the differentiated daughter TA cell going on to eventually become part of a fully differentiated tissue or organ system. Normal SSC are encouraged to undergo asymmetric divisions upon CBP/β-catenin antagonism and thus are not depleted from their niche. **Middle panel** Cancer stem cells (CSC) have a preference for symmetric divisions, thus leading to an increase in CSC in the niche over time with increased mutational burden. **Lower panel** CBP/β-catenin antagonists (ICG-001 or C82) induce symmetric differentiative divisions of the CSC population thereby stochastically clearing CSC from their niche.

Taking advantage of an intrinsic and critical difference between CSC and SSC, CBP/β-catenin antagonists enforce either symmetric (in CSC) or asymmetric (in SSC) differentiative cell division, providing efficacy and safety. Due to various mutations (e.g., p53, PTEN, etc.), CSC preferentially divide symmetrically whereas SSC divide asymmetrically [29,73,74]. CBP/β-catenin antagonists can thereby stochastically eliminate CSC via forced symmetric differentiation, whereas SSC in their niche divide asymmetrically and are maintained (Figure 5).

### 2.9. Pleiotropic Effects of CBP/β-Catenin Antagonists

ICG-001 was initially identified as a TCF/β-catenin antagonist that modulated Wnt signaling by selectively blocking the recruitment of the Kat3 coactivator CBP. However, after 25 years of research, it is now clear that effects of CBP/β-catenin antagonists involve far more than classical Wnt target genes and are highly pleiotropic. β-catenin, independent of TCFs, recruits CBP and p300 to enhancers (E) and super-enhancers (SE). Super-enhancers constitute a subset of enhancers that regulate genes controlling cellular identity and lineage fidelity [184,185,186,187,188]. Acetylated H3K27 (H3K27Ac), which is uniquely acetylated by Kat3 coactivators, demarcate E and SE [184,189,190,191,192,193]. The N and C-terminal intrinsically disordered regions (IDR) of β-catenin, independent of TCF/LEF interactions, can recruit either CBP or p300 into E/SE-driven complexes [194]. Already more than 20 years ago, differential Kat3 recruitment, with a predominant bias toward p300-mediated regulation to licensed enhancers, was noted [195]. CBP/p300 acetylation at enhancers simultaneously promote transcription initiation and elongation via pre-initiation complex (PIC) formation and RNAPII recruitment, independent of their roles in BRD4-dependent pause release [196]. The formation of the mouse cardiovascular system, lung and the small intestine are strongly impaired by p300 HAT mutation but significantly less by similar mutation of CBP, despite their highly conserved HAT domains [197]. This confirms the differential roles of CBP and p300 at critical E/SE during development and the critical role of p300 HAT activity during organogenesis. Experiments on human myoblast differentiation [157], cellular senescence [198], immune cell function [199] and in mouse embryonic fibroblasts at the single-cell genome-wide level [200], further attest to differential roles for Kat3 coactivators at E and SE.

SE in stem cells, provide for rapid fate switching when the niche local environment is modified [201], playing critical roles in development and disease [186,202]. Terminal transcription factors of the Wnt (i.e., TCF/LEF), TGF-β (Smad3), and LIF (Stat3) pathways often occupy SE and transcriptionally control stem cell states and can promote tumorigenesis [203,204]. Small molecule CBP/β-catenin antagonists by targeting ‘Wnt/β-catenin’-driven oncogenic ‘stemness’ and correcting lineage infidelity via SE modulation, provide an opportunity to target drug resistant CSC [205].

The CBP/β-catenin antagonist ICG-001, in diffuse intrinsic pontine gliomas, down-regulates genes involved in stemness maintenance (i.e., ID1 and ID3) by targeting SE, yet increase the expression of invasion promoting genes, via p300 recruitment, which can be effectively targeted by JQ-1, a BET inhibitor [206].

**Figure 6:**
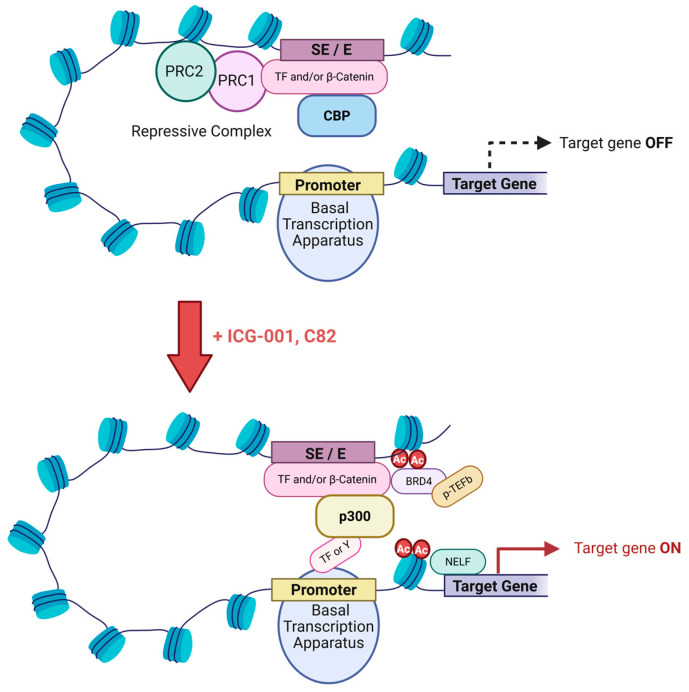
Intrinsically disordered regions (IDRs) of β-catenin connect transcription factor (TF)-interacting domains with the N-termini of either CBP or p300 in enhancer (E) and super-enhancer (SE) loci to interpret extracellular information and complex signaling cascades to orchestrate cell specific responses. The model depicts a small molecule specific CBP/β-catenin antagonist (ICG-001 or C82) dismissing CBP occupancy from a repressive complex with recruitment of p300 enhancing the assembly of tissue specific E/SE with pleiotropic effects on differentiation, lineage identity and fidelity, coupled to cellular metabolism.

CBP and p300 are seen as ‘molecular interpreters that can parse and/or conjugate the regulatory words, phrases, and sentences of the genome’ [207]. The amino terminal intrinsically disordered regions (IDR) of both Kat3 coactivators allows for promiscuous interaction with hundreds of cellular transcription factors, via their own IDRs to ‘interpret’ the gene encoded regulatory language. Within distinct tissue environments, differential activation of a common enhancer repertoire and the expression of divergent secondary transcription factors (TFs) that collaborate with core TFs, can establish tissue-specific enhancers [208]. β-catenin IDRs, via SE occupancy and interactions with both TFs and the N-termini of either Kat3 coactivator, assists in interpreting signal transduction cascades and extracellular information required for cell-specific responses [209,210,211,212,213]. In addition to canonical TCF/LEF family members [214,215,216,217,218,219], β-catenin interacts with a large number of TFs with assemblage of tissue-specific SEs fostered by the recruitment of either CBP or p300 [205] (Figure 6).

Treatment of pancreatic cancer cells with ICG-001 demonstrated wide ranging effects on super-enhancers and chromatin architecture over particularly broad epigenomic domains based on Hi-C analyses [220,221]. Insulin signaling genes were enriched in the altered chromatin structure and insulin signaling chromatin loops were significantly weakened by ICG-001, with strongly diminished IRS1 looping, in these cells [222].

## 3. Conclusions

Blocking recruitment of the N-terminal of CBP to various E and SE, with or without a corresponding increase in p300 recruitment, and the de novo formation of many new E/SE, broadly affects stem cell differentiation, lineage commitment, hypoxia, immune cell function, metabolism, cellular senescence, etc., leading to the wide array of pleiotropic effects observed [22,29,223,224]. Additionally, selective disruption of CBP recruitment via its very N-terminal domain with small molecule CBP/β-catenin antagonists, frees up the limited amount per cell of CBP for recruitment to new E/SE, via alternative domains (e.g., KIX domain), to drive differentiative programs [225,226] (Figure 6). Wnt signaling cascade intrinsic complexity and further crosstalk with multiple pathways, clearly represents a major obstacle to safe therapeutic targeting [22,29]. Specific small molecule CBP/β-catenin antagonists can safely eliminate CSC via forced symmetric differentiation, thereby sensitizing resistant tumors to conventional or immunotherapy. Preclinically CBP/β-catenin antagonists by targeting CSC, dramatically sensitized tumors to chemotherapy and prevented secondary tumor engraftment, however, they did not affect tumor growth [171]. Therefore, unsurprisingly, in the PRI-724 Phase I clinical study, no objective RECIST criteria responses were seen, despite on-target effects in circulating tumor cells, a surrogate “stem cell” population [181]. Furthermore, PRI-724’s excellent safety profile and promising on target clinical effects both in oncology and liver fibrosis bode well for specific CBP/β-catenin antagonist development, to maintain or regain lineage fidelity, commonly defective in diseases of aging including cancer, fibrosis and neurodegeneration. Intriguingly, *survivin/Birc5* expression has been shown to be critical for both hES cell generation of teratomas [227] and oncogene targeted stem cell initiation of basal cell carcinoma [228]. The excellent safety profile of CBP/beta-catenin antagonists coupled with their capacity to down-regulate *survivin*/*Birc5* expression auger well for the prospects of utilizing CBP/beta-catenin antagonists to prevent malignancies.

## Data Availability

No new data was generated for the preparation of this manuscript. All data from the authors lab that is discussed is available in the primary references cited in the text.

## Abbreviations

CREBBP/CBP	CREB Binding Protein
SSC	Somatic stem cell
CSC	Cancer stem cell
LSC	Leukemic stem cell
CTC	Circulating Tumor Cell
E/SE	Enhancer/Super-Enhancer
TME	Tumor Microenvironment
CHIP	Clonal Hematopoiesis of Indeterminate Potential
FAO	Fatty Acid Oxidation
OXPHOS	Oxidative Phosphorylation
MC1	Mitochondrial Complex 1
ROS	Reactive Oxygen Species
